# WRKY Transcriptional Factor *Il*WRKY70 from *Iris laevigata* Enhances Drought and Salinity Tolerances in *Nicotiana tabacum*

**DOI:** 10.3390/ijms242216174

**Published:** 2023-11-10

**Authors:** Gongfa Shi, Guiling Liu, Huijun Liu, Nuo Xu, Qianqian Yang, Ziyi Song, Wangbin Ye, Ling Wang

**Affiliations:** College of Landscape Architecture, Northeast Forestry University, Harbin 150040, Chinaliuguiling90@126.com (G.L.); xunuo@nefu.edu.cn (N.X.); 16650601598@nefu.edu.cn (Q.Y.); lgl@nefu.edu.cn (W.Y.)

**Keywords:** *Iris laevigata*, WRKY transcription factor, salt stress, drought stress

## Abstract

Drought and high salinity greatly affect plant growth and development. WRKY transcription factors play a key role in plant tolerance to abiotic stress, but the functions of *WRKY*s in the ornamental monocotyledon *Iris laevigata* remain largely unexplored. In this study, we cloned *IlWRKY70* and found that it is a Group III WRKY localized in the nucleus. The expression of *IlWRKY70* was induced by NaCl and PEG-6000, which reached peaks (4.38 and 5.65 times) after 3 h and 1 h, respectively. The exogenous overexpression of *IlWRKY70* in *N*. *tabacum* significantly improved the resistance under NaCl and drought treatments, as evidenced by higher germination rates, longer root lengths, and increased fresh weights compared to those of control plants. In addition, transgenic seedlings showed significantly reduced wilting, higher photosynthetic performance, higher Fv/Fm and chlorophyll content, and lower stomatal conductance. Moreover, transgenic lines showed higher antioxidant enzymatic activities, lower reactive oxygen species (ROS), and lower malondialdehyde contents. Accordingly, we also found higher expressions of antioxidant defense genes, including *SOD*, *CAT*, and *POD*, in transgenic lines compared to controls under salt and drought stresses. Thus, *IlWRKY70* enhances the abilities of salt and drought tolerances in plants, at least partially, via ROS regulation and can be used for breeding *I. laevigata* possessing enhanced salt and drought resistances.

## 1. Introduction

Salt and drought stresses are among the most prevalent abiotic stresses in the plant kingdom [1]. Recent years have witnessed notable shifts in climate dynamics, resulting in altered rainfall patterns, thereby exacerbating land desertification [2] and salinization [3] trends. It is noteworthy that both salt and drought conditions predominantly induce osmotic stress in plants. Beyond water-scarcity-induced drought, salt-induced osmotic pressure also engenders water deficits in plants, compounded by the direct toxicity of salt ions [4]. Therefore, it is very important to screen and study plant salt- and drought-resistance genes, clarify the mechanisms of plant salt and drought resistances, and use genetic engineering to quickly create various salt- and drought-resistant plants to solve practical problems. *I. laevigata* is a perennial cold-resistant herb of the Iris genus. Its flowers are blue and purple, large and colorful, and tall and beautiful, with a long flowering period. It grows mostly in wetlands and swamps and has high ornamental value. The characteristic of *I. laevigata*’s moisture preference and the increasing salinization of urban land severely limit the promotion and application of *I. laevigata* outside of wetlands. Therefore, exploring genes that regulate both salt and drought resistances in plants and creating *I. laevigata* varieties which can possess drought and salt resistances through genetic engineering can greatly expand the application scenarios of *I. laevigata*, which is of great significance for enriching cold landscapes.

Numerous studies have demonstrated that plants subjected to abiotic stress transmit stress signals to stress-response transcription factors (TFs) via signal transduction pathways, regulating downstream resistance-related genes to combat adverse environmental damage. Notably, transcription-factor families, such as bZIP, bHLH, WRKY, and MYB, are involved in plant responses to abiotic stress [5,6,7,8]. Among them, the WRKY family is one of the largest transcription-factor families unique to plants, and the WRKY protein has a fast response to adverse environments [9], playing a key role in the tolerance of various plants to abiotic stress. For example, *LlWRKY22* promotes the heat tolerance of lilies through the self-activation and activation of *LlDREB2B* [10], *PyWRKY75* enhances the cadmium stress tolerance of poplar trees [11], *GmWRKY21* of soybeans enhances the tolerance of arabidopsis to aluminum stress [12], while *WRKY8* of tomatoes can promote plant stress resistance under drought and salt stresses [13].

All WRKY proteins derive their nomenclature from the presence of conserved WRKY domains (WDs), which are characterized by the medium to high conservation of the WRKYGQK motif and C2H2 or C2HC zinc finger motifs. These zinc finger motifs play a pivotal role in binding specifically to the W-box structure within the target genes, thereby orchestrating the regulation of diverse target genes [9,14]. The WRKY family, classified based on the number of conserved WRKY domains and the type of zinc finger structure, comprises four distinct groups [15]. The WD of Group I has two C2H2 zinc finger structural motifs, the WD of Group II has one C2H2 zinc finger structural motif, the WD of Group III has one C2HC zinc finger structural motif, and the WD of Group IV lacks a zinc finger structural motif [9]. It is widely believed that Group I is the ancestral group, with Group II evolving subsequently through the loss of one WRKY domain. The emergence of Group III can be attributed to a mutation in the C-terminal zinc finger of Group II. Research indicates that Group III evolved relatively late in the evolutionary timeline of terrestrial plants and is unique to higher plants [16,17].

Although the functional role of WRKY genes has primarily been explored in crops [9], the utilization of WRKY genes in ornamental flowers, particularly within the monocotyledonous plant *I. laevigata*, has been relatively unexplored. One notable member of the Group III WRKY transcription factors is WRKY70, which has garnered attention in recent years for its ability to confer drought tolerance, salt tolerance, and disease resistance in plants [18,19,20,21,22]. However, the role of *WRKY70* in drought and salt tolerances has only been studied in dicotyledons, and *WRKY70* from different sources may have opposite effects on drought or salt tolerance. *WRKY70* from a special group of monocot plants has not been reported. *I. laevigata* is a poorly understood but magnificent monocotyledon ornamental plant with no reported genes; and as a hygrophyte, it is necessary to open the study of drought- and salt-stress-resistance genes.

In the context of this study, we have successfully cloned the *WRKY70* gene from *I. laevigata*, which demonstrates inducible expression in response to salt and drought stress conditions. The overexpression of *IlWRKY70* has been shown to significantly enhance salt and drought tolerances in transgenic tobacco seeds and seedlings. This discovery positions *IlWRKY70* as a promising candidate gene for improving drought and salt resistances not only in *I. laevigata* but also in other iris species, with potential implications for the advancement of ornamental horticulture. It also provides a basis for further study on the function of *WRKY70* in other monocot plants.

## 2. Results

### 2.1. IlWRKY70 Gene Cloning and Phylogenetic Tree Analysis

**Figure 1:**
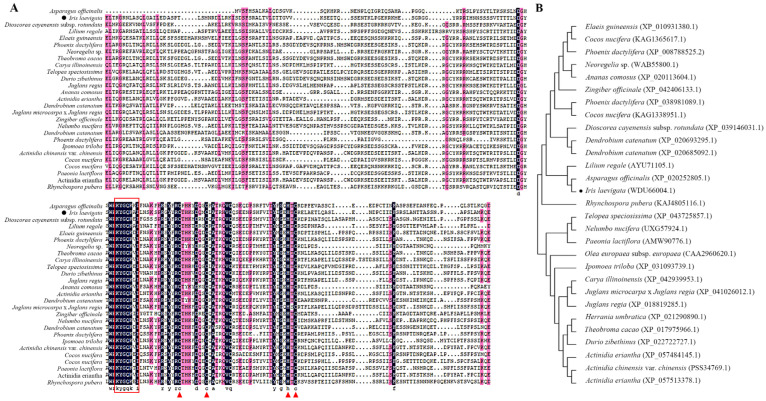
Sequence analysis and phylogenetic tree analysis of *Il*WRKY70. (**A**) Evolutionary tree of *Il*WRKY70 and other plant WRKY70. The red box is the highly conserved domain “WRKYGQK” of the WRKY family. The pink background color represents >75% similarity. The black background color represents 100% agreement. The red triangle indicates WRKY’s zinc finger structure. (**B**) Evolutionary tree of *Il*WRKY70 and its homologs. Black dots indicate IlWRKY70 protein from *I. laevigata*.

The 900 bp *IlWRKY70* gene, which encodes 299 amino acids, was cloned from *I. laevigata* cDNA through PCR amplification. ProtParam analysis revealed that the *Il*WRKY70 protein has a molecular weight of 32.866 kDa, a theoretical isoelectric point of 5.01, total numbers of negatively charged residues (Asp + Glu) of 42 and positively charged residues (Arg + Lys) of 30, and an instability index (II) of 49.34, indicating it is an unstable protein. According to ProtScale analysis, the highest hydrophilicity score for the *Il*WRKY70 protein was at amino acid position 157 (−2.656), while its highest hydrophobicity score was at position 233 (1.667). The number and scores of hydrophilic amino acids in the peptide chain were higher than those for hydrophobic ones, suggesting that the *Il*WRKY70 protein is hydrophilic. By constructing the WRKY70 phylogenetic tree with other species, *Il*WRKY70 was the most homologous to the WRKY70 protein of *Asparagus officinalis* in the monocotyledon plant (Figure 1B). Through protein sequence alignment (Figure 1A), the highly conserved domain WRKYGQK [9] in the WRKY family was also identified in the *Il*WRKY70 protein sequence, and the assumed C2HC zinc finger structure of C-X7-C-X23-H-X-C was identified in this domain.

### 2.2. Expression of IlWRKY70 in I. laevigata

**Figure 2:**
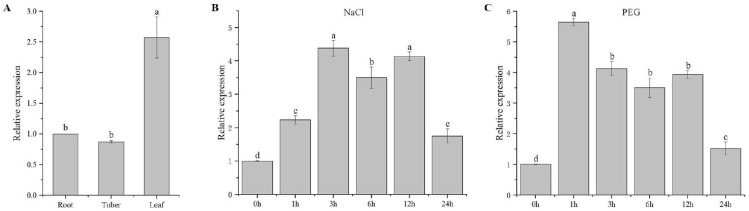
Expression analysis of *IlWRKY70*. (**A**) Expression analysis of *IlWRKY70* in roots, tubers, and leaves of *I. laevigata*. (**B**) Expression analysis of *IlWRKY70* in response to NaCl stress at different times. (**C**) Expression analysis of *IlWRKY70* in response to PEG-6000 stress at different times. The different letters on the top of the bar chart are significantly different from each other (*p* < 0.05).

The expression of *IlWRKY70* was examined in the roots, tubers, and leaves of *I. laevigata*, and the results showed that the expression level of *IlWRKY70* in leaves was significantly higher than those in roots and tubers (Figure 2A). The expression of *IlWRKY70* increased significantly after treatment (Figure 2B,C) and reached the peak values (4.38 times) after 3 h of salt treatment and (5.65 times) after 1 h of drought-stress treatment.

### 2.3. Subcellular Localization of IlWRKY70

**Figure 3:**
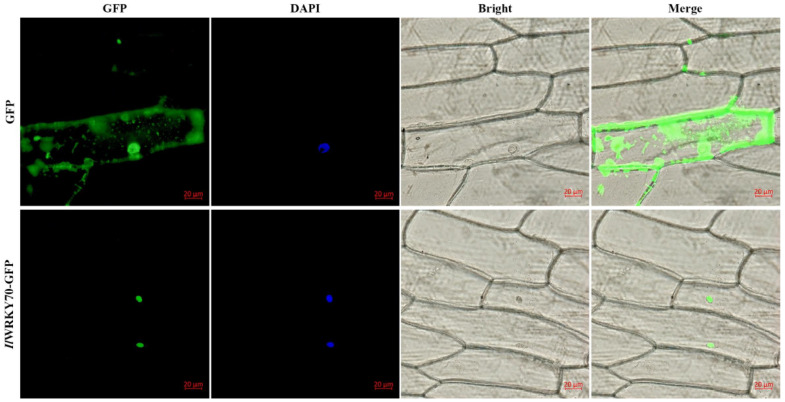
Subcellular localization of *Il*WRKY70 protein. pCAMBIA1300:GFP and pCAMBIA1300:*IlWRKY70*-*GFP* were transfected into onion epidermal cells; and after DAPI staining, they were observed by fluorescence microscopy using 395 nm excitation GFP and 358 nm excitation DAPI nuclear localization dye, respectively. From left to right, green fluorescence of GFP, blue light of DAPI nuclear localization dye, cells under bright field, and cells under superposition of GFP and bright field. The red bar represents a scale bar of 20 μm.

To determine the subcellular localization of the *Il*WRKY70 protein, the pCAMBIA1300:*IlWRKY70-GFP* plasmid vector was constructed and transiently transformed into onion epidermal cells. With the help of fluorescence microscopy, DAPI staining showed the location of the nucleus. The fluorescence signal of the empty vector pCAMBIA1300-*GFP* protein was present in both the cytoplasm and the nucleus, while the fluorescence signal of the *Il*WRKY70 protein was only localized in the nucleus (Figure 3).

### 2.4. Establishment of Transgenic Tobacco Lines Overexpressing IlWRKY70

**Figure 4:**
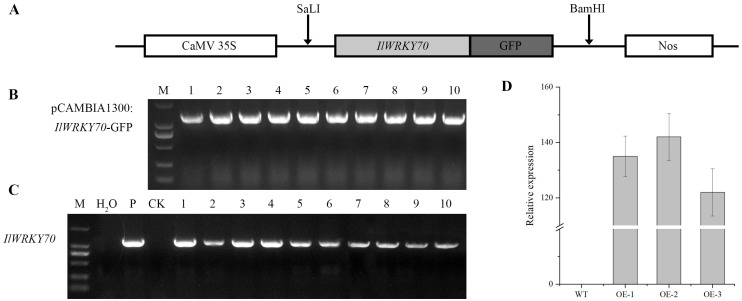
Establishment of *IlWRKY70* transgenic tobacco lines. (**A**) Schematic representation of the pCAMBIA1300:*IlWRKY70*-*GFP* vector construction. (**B**) PCR validation gel plot of the successful construction of pCAMBIA1300:*IlWRKY70*-*GFP* vector; M is marker of length 2000. The numbers 1-10 represent a positive plasmid. (**C**) Positive validation gel plot of *IlWRKY70* transgenic T0 generation plants; M is marker of length 2000, H_2_O is negative control, P is pCAMBIA1300:*IlWRKY70*-*GFP* plasmid positive control, CK is WT tobacco, and 1–10 is T0 transgenic tobacco. (**D**) Relative expression of *IlWRKY70* in three T3 transgenic tobacco lines.

The ORF sequence of *IlWRKY70* was successfully cloned into the pCAMBIA1300:*IlWRKY70*-*GFP* plasmid vector through homologous recombination (Figure 4A,B). Ten T0 transgenic tobacco lines were successfully transformed using *Agrobacterium tumefaciens*-mediated leaf disc transformation (Figure 4C). The positive inbred progenies were screened with hygromycin B and identified by PCR until the T3 generation. After that, three positive lines were randomly selected and named as overexpression line 1 (The abbreviation is OE-1, and the others are similar), OE-2, and OE-3 positive seedlings for subsequent experiments. RT-qPCR analysis showed that the *IlWRKY70* gene was highly expressed in OE-1, OE-2, and OE-3 compared to the wild type (WT) (Figure 4D).

### 2.5. Physiological Indexes of Overexpressed N. tabacum Seeds

**Figure 5:**
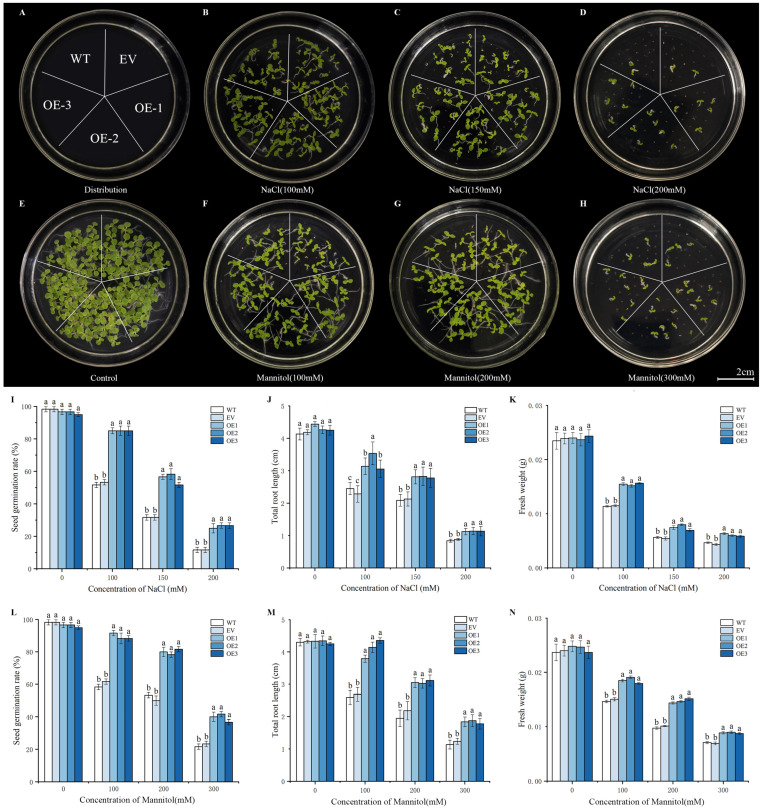
Overexpression of the *IlWRKY70* gene enhanced the response of tobacco seeds to salt and drought stresses. (**A**) Schematic diagram of tobacco seed zoning for different lines. The white horizontal line in the figure represents 2 cm. (**B**–**D**) Germination and growth of tobacco seeds from WT, EV, and OE lines after 15 days of different salt stress treatments. (**E**) Germination and growth of tobacco seeds from WT, EV, and OE lines after 15 days in MS medium. (**F**–**H**) Germination and growth of tobacco seeds from WT, EV, and OE lines after 15 days of different drought stress treatments. (**I**–**K**) Germination rate, total root length, and fresh weight of tobacco seeds in each line treated with different gradients of salt stress for 15 days. (**L**–**N**) Germination rate, total root length, and fresh weight of tobacco seeds in different lines treated with different gradients of drought stress for 15 days. The letters above the bar chart represent significant differences between each other, *p* < 0.05.

To explore the effect of the exogenous *IlWRKY70* expression on salt and drought stresses, the tobacco seeds of the OE-1, OE-2, and OE-3 lines overexpressing *IlWRKY70* at the T3 generation were treated with salt stress or drought stress for 15 days, with WT and EV seeds as controls (Figure 5A–H). Under MS medium conditions, all the tobacco seeds germinated normally and grew well after 15 days (Figure 5E), indicating that there was no significant difference between transgenic plants, empty vector, and wild type under normal conditions. However, after three gradient treatments of salt stress (NaCl concentrations of 100, 150, and 200 mM) and drought stress (mannitol concentrations of 100, 200, and 300 mM), the seed germination rate (Figure 5I,L), total root length (Figure 5J,M), and fresh weight (Figure 5K,N) of the OE-1, OE-2, and OE-3 transgenic lines were significantly higher than those of the WT and EV lines (*p* < 0.05), and there was no significant difference between the WT and EV lines (*p* > 0.05).

### 2.6. Physiological Indices of Overexpressed N. tabacum Seedlings

**Figure 6:**
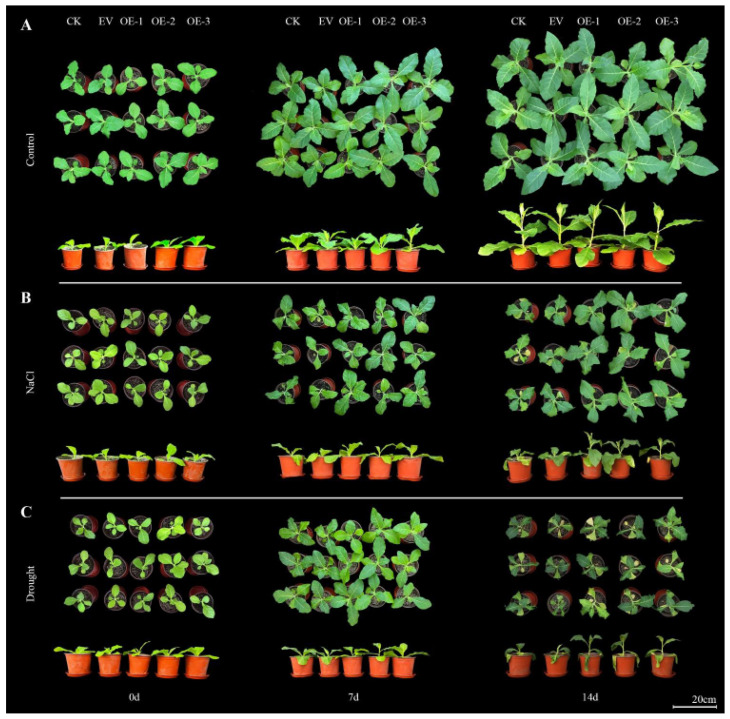
Phenotypic changes in tobacco seedlings overexpressing the *IlWRKY70* gene under salt and drought stresses. (**A**) Top view and elevation of the growth status of WT, EV, and OE lines of tobacco at 0, 7, and 14 days in the normal growth state. (**B**) Top view and elevation of the growth status of WT, EV, and OE lines of tobacco at 0, 7, and 14 days under NaCl stress at 300 mM. (**C**) Top view and elevation of the growth status of WT, EV, and OE lines of tobacco at 0, 7, and 14 days under natural drought stress. The scale bar is 20 cm.

The transgenic tobacco seedlings were subjected to natural drought and salt stresses (NaCl concentration of 300 mM) to assess the resistance level. Under normal growth conditions, CK, EV, OE-1, OE-2, and OE-3 exhibited similar growth patterns with no significant differences among the five lines (Figure 6A). However, on the seventh day of salt stress, WT and EV showed lower leaf growth rates compared to the transgenic lines and displayed evident wilting symptoms. At day 14, the transgenic lines demonstrated significantly higher leaf growth rates and plant heights than the WT and EV lines, whereas WT and EV plants wilted, with yellowing signs on the lower leaves along with a slower overall growth rate (Figure 6B). Similarly, on the seventh day of drought stress, both WT and EV plants exhibited obvious wilting symptoms. At day 14, although WT and EV plants exhibited clear wilting throughout their foliage, transgenic plants displayed noticeable wilting on the lower leaves but only slight wilting on the upper leaves along with significantly greater heights compared to those WT and EV plants (Figure 6C).

**Figure 7:**
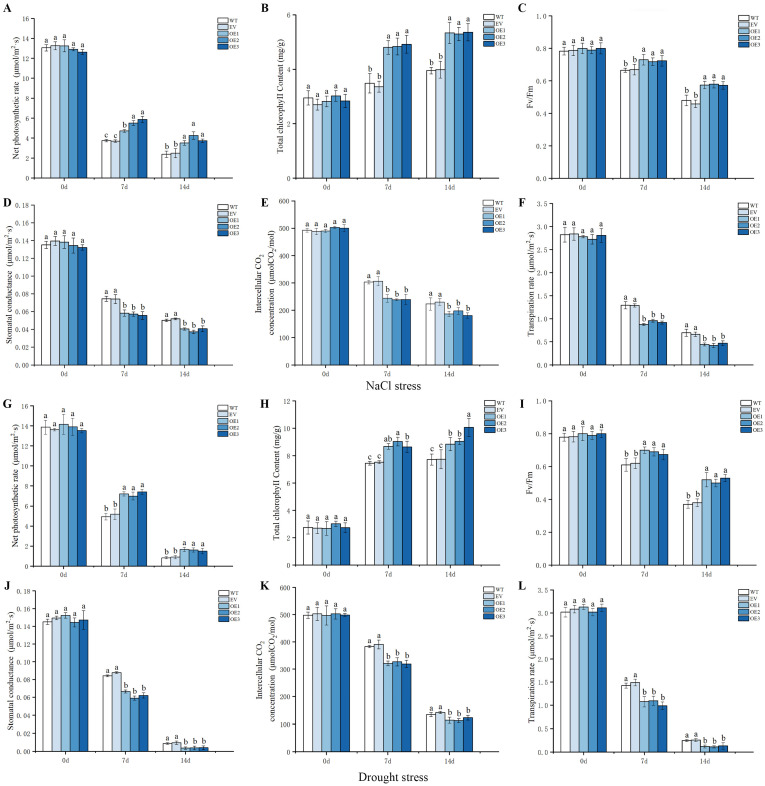
Analysis of various photosynthetic and transpiration rate indexes of tobacco seedlings overexpressing *IlWRKY70* under salt and drought stresses. (**A**–**F**) Net photosynthetic rate, total chlorophyll content, Fv/Fm, stomatal conductance, intercellular CO_2_ content, and transpiration rate of WT, EV, and OE tobacco lines at 0, 7, and 14 days under 300 mM NaCl stress. (**G**–**L**) Net photosynthetic rate, total chlorophyll content, Fv/Fm, stomatal conductance, intercellular CO_2_ content, and transpiration rate of WT, EV, and OE tobacco lines under natural drought stress at 0, 7, and 14 days. The letters above the bar chart represent significant differences between each other, *p* < 0.05.

Plants use H_2_O and CO_2_ to produce organic matter through photosynthesis and maintain their growth. The net photosynthetic rate, total chlorophyll content, Fv/Fm, stomatal conductance, and intercellular CO_2_ content are common indicators reflecting the level of a plant’s photosynthetic capacity. Higher values of these indicators tend to represent higher photosynthetic capacities of plants. The transpiration rate can reflect the water demand of the plant, and the faster the transpiration rate, the lower the water demand of the plant. To explore the photosynthetic capacity and growth status of transgenic tobacco under salt and drought stresses, the above six indicators were measured. The results showed that at day 0 of the salt stress, there was no significant difference among all the six indexes (Figure 7A–F). With increasing salt stress time, the total chlorophyll content showed an increasing trend (Figure 7B), but the net photosynthetic rate, Fv/Fm, stomatal conductance, intercellular CO_2_ content, and transpiration rate (Figure 7A,C–F) showed a decreasing trend. At 7 and 14 days, the net photosynthetic rate, total chlorophyll content, and Fv/Fm of the three *IlWRKY70* overexpressing tobacco lines were significantly higher than those of the WT and EV lines (*p* < 0.05), and the stomatal conductance, intercellular CO_2_ content, and transpiration rate were significantly lower than those of the control lines (*p* > 0.05). Similarly, at day 0 of the drought stress, there was no significant difference (Figure 7G–L) in the six indexes of all the strains (*p* > 0.05). With the extension of the stress time, the total chlorophyll showed an increasing trend (Figure 7H), while the net photosynthetic rate, Fv/Fm, stomatal conductance, intercellular CO_2_ content, and transpiration rate (Figure 7G,I–L) showed a decreasing trend, indicating that the photosynthetic capacity of the plants was severely limited under the drought stress. With increasing drought time, the net photosynthetic rate, stomatal conductance, and plant transpiration rate decreased rapidly; all reached very low levels at 14 days; and the plants were severely short of water. The net photosynthetic rate, total chlorophyll content, and Fv/Fm of the *IlWRKY70* overexpressed tobacco lines were significantly higher than those of the WT and EV lines at 7 and 14 days, while the stomatal conductance, intercellular CO_2_ content, and transpiration rate were significantly lower than those of the control lines. These results indicated that under salt stress and drought stress, *IlWRKY70* alleviated the decrease in photosynthetic efficiency by increasing or maintaining the total chlorophyll content, and increased the plants’ water resistance under osmotic stress by closing the stomata to retain water.

Plants under abiotic stress often suffer from oxidative stress and produce excessive oxygen free radicals (ROS), which cause damage to plants. ROS and lipids undergo peroxidation, ultimately producing the cytotoxic substance malondialdehyde (MDA), which endangers plant growth. Superoxide anions ($O_{2}^{-}$) and H_2_O_2_ are the main ROS substances [13]. Superoxide dismutase (SOD), catalase (CAT), and peroxidase (POD) are the main enzymes in the plant antioxidant system, effectively removing H_2_O_2_ and other substances produced by stress [23]. To explore the resistance levels of tobacco overexpressing *IlWRKY70* under salt stress and drought stress, the WT and EV lines were selected as controls to detect the SOD, CAT, and POD enzymatic activities and the contents of MDA, $O_{2}^{-}$, and H_2_O_2_ in tobacco leaves at 0, 7, and 14 days. The leaves were stained with NBT and DAB to show the accumulation of $O_{2}^{-}$ and H_2_O_2_ in the leaves, respectively.

**Figure 8:**
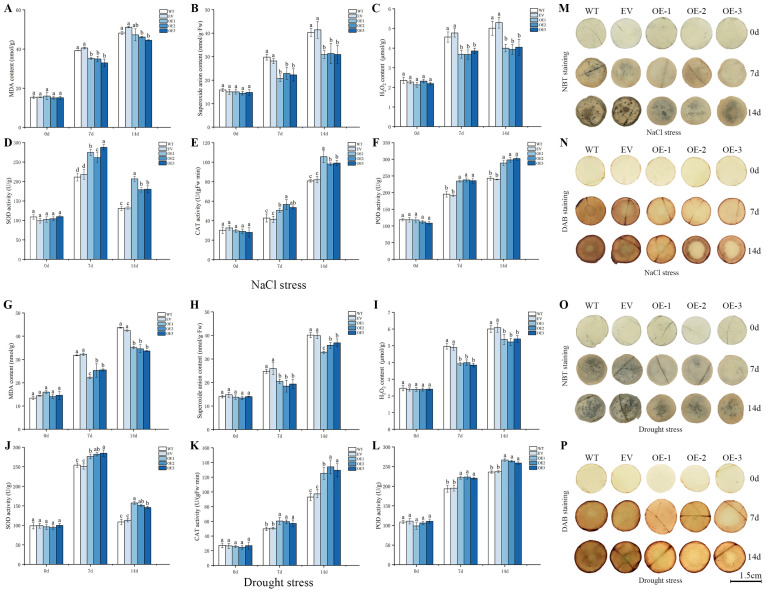
Analysis of physiological indexes of tobacco seedlings overexpressing *IlWRKY70* under salt stress and drought stress. (**A**–**F**) The contents of MDA, $O_{2}^{-}$, H_2_O_2_ and the activities of SOD, CAT, and POD in WT, EV, and OE tobacco leaves were measured at 0, 7, and 14 days after 300 mM NaCl stress. (**G**–**L**) The contents of MDA, $O_{2}^{-}$, H_2_O_2_ and the activities of SOD, CAT, and POD in the leaves of the WT, EV, and OE lines were measured at 0, 7, and 14 days of natural drought stress. Different letters on the bar chart represent significant differences, *p* < 0.05. (**M**,**N**) Plots of leaf disc staining using NBT and DAB in WT, EV, and OE lines at 0, 7, and 14 days of NaCl stress at 300 mM. (**O**,**P**) Plots of leaf disc staining using NBT and DAB in WT, EV, and OE lines at 0, 7, and 14 days of natural drought stress. Deeper NBT staining means more $O_{2}^{-}$ accumulation, deeper DAB staining means more H_2_O_2_ accumulation, and more $O_{2}^{-}$ and H_2_O_2_ accumulation means more severe plant damage. The black horizontal line in the figure represents 1.5 cm, the diameter of the leaf disc.

The results showed that there was no significant difference in the contents of MDA, $O_{2}^{-}$, and H_2_O_2_ among all the plants at day 0 of the salt stress (Figure 8A–C). With the number of stress days increases, the contents of these three components showed an upward trend, indicating that the plants were gradually subjected to enhanced oxidative stress damage. The contents of MDA, $O_{2}^{-}$, and H_2_O_2_ in the three lines overexpressing *IlWRKY70* on days 7 and 14 were significantly lower than those in WT and EV (*p* < 0.05). Similarly, the contents of MDA, $O_{2}^{-}$, and H_2_O_2_ in all the lines were not significantly different on day 0 of the drought stress (Figure 8G–I). However, the levels of these substances increased in all the lines on days 7 and 14 under drought stress conditions. The contents of the three substances in the overexpressed tobacco were significantly lower than those in the WT and EV lines (*p* < 0.05). The NBT and DAB staining results also visually demonstrated that the *IlWRKY70* overexpressed tobacco accumulated less $O_{2}^{-}$ and H_2_O_2_ under salt and drought stresses than the WT and EV plants (Figure 8M–P). The results showed that the enzymatic activities of SOD, CAT, and POD were almost the same at day 0 of the salt stress (Figure 8D–F). However, with the number of stress days increases, the enzymatic activities of SOD, CAT, and POD in the OE-1, OE-2, and OE-3 lines were significantly higher than those in the WT and EV lines on days 7 and 14 (*p* < 0.05). Consistently, under drought stress, there was no significant difference in the three enzymatic activities at the initial stage of the stress (Figure 8J–L), but with the number of stress days increases, the three enzymatic activities of the OE-1, OE-2, and OE-3 lines at 7 and 14 days were significantly higher than those of the WT and EV lines (*p* < 0.05). These results indicated that the tobacco overexpressing *IlWRKY70* had a stronger ability to remove ROS and accumulate fewer harmful substances under salt and drought stresses than the control and could better cope with salt and drought stresses.

### 2.7. The Effect of the Overexpressed IlWRKY70 on the Expression Levels of Stress-Related Genes NtSOD, NtCAT, and NtPOD under Salt Stress and Drought Stress

**Figure 9:**
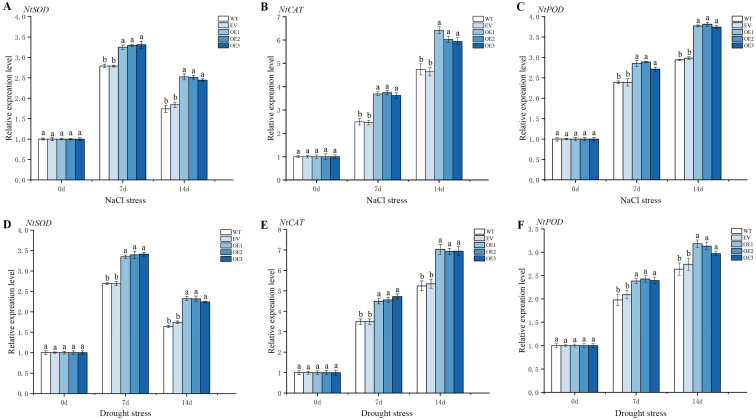
Expression analysis of genes related to salt stress and drought stress in over-expressed *IlWRKY70* tobacco. (**A**–**C**) Real-time expression of *NtSOD*, *NtCAT*, and *NtPOD* genes in WT, EV, and OE tobacco lines at 0, 7, and 14 days of NaCl stress at 300 mM. (**D**–**F**) Real-time expression of *NtSOD*, *NtCAT*, and *NtPOD* genes in tobacco leaves of WT, EV, and OE lines at 0, 7, and 14 days of drought stress. Different letters on the bar chart represent significant differences, *p* < 0.05.

RT-qPCR was used to analyze the changes in the expression levels of stress-responsive genes after overexpressing *IlWRKY70* in tobacco. After 7 and 14 days of salt stress (NaCl concentration of 300 mM) and natural drought stress, the expression levels of the *NtSOD*, *NtCAT*, and *NtPOD* genes in the overexpressed *IlWRKY70* tobacco were significantly higher than those in the WT and EV lines (Figure 9), indicating that *IlWRKY70* could directly or indirectly regulate the expression of downstream stress-responsive genes.

## 3. Discussion

An increasing number of studies has shown that WRKY transcription factors play a crucial role in plants’ responses to biotic or abiotic stress. Recent studies have shown that different genes within the WRKY family play an important role in improving plants’ resistances to disease [24,25], salt [26], and drought [27]. However, studies on the resistance functions of these *WRKY* genes have mostly concentrated on crop plants [13,28] and have rarely been explored in ornamental flowers, especially in the monocotyledonous plant *I. laevigata*.

This study cloned and identified the *WRKY70* gene of *I. laevigata*, which has the highest homology with the WRKY70 protein of *Asparagus officinalis* in monocotyledonous plants. *IlWRKY70* has a classic WRKY domain and a C2HC zinc finger structure, belonging to the Group III WRKY family. *IlWRKY70* is in the nucleus, consistent with the reported localization of its homologous genes, indicating its ability to act as a transcription factor. WRKY70 has been confirmed to be involved in plant drought and salt stress responses. However, in early studies, *WRKY70* was considered as a negative regulatory factor for salt and drought stresses. In *Populus simonii* × *P. nigra*, there are many cis-acting elements in the *PsnWRKY70* promoter that respond to biotic and abiotic stresses, and *PsnWRKY70* has self-regulation ability. Under salt stress, the expression of *PsnWRKY70* is downregulated in both wild and overexpressing poplar trees. Poplar plants that inhibit *PsnWRKY70* have stronger salt resistance [21]. Through research in arabidopsis, the *WRKY70* single mutant and the *WRKY70* and *WRKY54* double mutants have better drought resistance, which may be achieved by regulating leaf stomatal closure [22]. However, in the *WRKY70* and *WRKY54* double mutants, the expression of 58 response genes to osmotic stress was strongly suppressed, and the proline content in the plant was significantly reduced. *WRKY70* may not only be a negative regulatory factor of abiotic stress but also has the potential to enhance osmotic stress. In recent years, this hypothesis has been confirmed because the *WRKY70* gene of *Myrothamnus flabellifolius* is significantly upregulated in the early dehydration stage, and the introduction of *WRKY70* to arabidopsis can significantly improve the salt and drought resistances of arabidopsis [19]. Meanwhile, in this study, the significant expression patterns of *IlWRKY70* under salt and drought stresses also indicate that it may play a key role in regulating the response of *I. laevigata* to salt and drought stresses.

In abiotic stress environments, the ability of seeds to germinate and grow normally is a prerequisite for plants to reproduce and survive in harsh environments. Plant roots are highly sensitive to salt and drought stresses [1], and the root length can indicate the underground growth status and resistance level of plants [29,30,31]. Transgenic lines treated with NaCl stresses at different gradients (0, 100, 150, and 200 mM) for 15 days and drought stresses at different gradients (mannitol concentrations of 0, 100, 200, and 300 mM) for 15 days showed higher germination rates, longer total root lengths, and higher fresh weights. This indicates that the overexpression of *IlWRKY70* improves the salt and drought resistances of transgenic tobacco seeds to a certain extent.

Plants provide the organic matter necessary for their own growth through photosynthesis, which is one of the most critical biological processes for plant survival [32]. In addition, salt and drought stresses cause plants to lose water owing to high osmotic pressure; this, in turn, leads to stomatal closure, reducing the intercellular CO_2_ content and blocking the supply of raw materials for plant photosynthesis [32,33]. The PSII primary light-energy-conversion efficiency (Fv/Fm) is commonly used to represent the maximum photosynthetic capacity of plants [34]. The tobacco seedlings overexpressed with *IlWRKY70* exhibited better growth statuses, higher net photosynthetic rates, higher chlorophyll contents, and higher PSII primary light-energy-conversion efficiencies under both salt and drought stresses. And overexpressing *IlWRKY70* tobacco plants have a lower stomatal conductance, lower intercellular CO_2_ content, and lower transpiration rate. This may be due to the smaller stomatal openings of transgenic plants under drought and salt stresses, reducing water evaporation and gas exchange and increasing the plant’s water retention capacity. This finding aligns with the research by Sun and Xiang et al., who demonstrated that the overexpression of *WRKY70* does not affect the degree of stomatal opening in plants under regular conditions. However, it has been shown that *WRKY70* overexpression can decrease the degree of stomatal opening during drought stress. [18,19]. The research results indicate that *IlWRKY70* can maintain more chlorophyll content to cope with salt and drought stresses and may reduce the degree of plant stomatal opening to maintain the water content of plants during the stress process. However, further research is needed on how *IlWRKY70* maintains or produces chlorophyll content and how it participates in plant stomatal regulation.

Plants produce a large amount of ROS substances, such as superoxide anions and H_2_O_2_, under various abiotic stresses [35]. ROS can damage plant cell membranes and produce toxins, such as MDA, that harm plant growth [36,37]. To prevent the excessive accumulation of ROS under stress, plant cells have evolved antioxidant mechanisms, such as enzymatic systems, including common antioxidant enzymes, such as SOD, CAT, and POD [23,38,39]. Tobacco seedlings overexpressing *IlWRKY70* exhibit less accumulation of MDA, superoxide anions, and H_2_O_2_ under salt and drought stresses while also exhibiting higher SOD, CAT, and POD enzymatic activities. Compared with the control plants, the transgenic tobacco possessed expression levels of genes related to ROS clearance under salt and drought stresses that were significantly increased, indicating that *IlWRKY70* may enhance the plant’s ability to scavenge ROS by increasing the expression of genes related to plant antioxidant enzyme systems in response to salt and drought stresses.

In terms of the responses of *WRKY70* to salt and drought stresses, previous studies have focused on *WRKY70* derived from dicot plants and found that *WRKY70* derived from different plants may have different effects on salt and drought stresses. *IlWRKY70* is the first *WRKY70* gene to be extracted from a monocotyledon ornamental plant species and demonstrated to be involved in the response of *I. laevigata* to drought and salt stresses. Although our indirect validation of *IlWRKY70*’s positive effect on drought and salt stresses only in tobacco has some limitations due to the lack of a genetic transformation system in the monocotyledon *I. laevigata*, it has pioneered the broadening of the range of sources of *WRKY70* research and is a good foundation for further research on drought and salt tolerance mechanisms in monocot plants. In the future, it is expected that *IlWRKY70* will be used or *WRKY70* will be obtained from very drought- and salt-tolerant *I. lactea* and *I*. *halophila* to improve the drought resistance of irises that grow in humid environments, such as *I. ensata*, and to improve the drought tolerances of varieties of *I. ensata* var. *hortensis* through crossbreeding. Of course, the resistance breeding of many famous monocot ornamental plants, may has a new foothold.

## 4. Materials and Methods

### 4.1. Plant Materials and Growth Conditions

*I. laevigata* plants were cultivated in the nursery at Northeast Forestry University’s College of Landscape Sciences. Onions for subcellular localization studies were procured from Carrefour Supermarket, while *N. tabacum* (ordinary tobacco) seeds were provided by the research group’s laboratory. The tobacco seeds were sterilized with 75% alcohol (LIRCON, Dezhou, China) on a sterile platform for 1 min, rinsed three times with sterile water, sterilized with 2% NaClO (XiLONG, Shenzhen, China) for 10 min, rinsed five times with sterile water, and then planted in MS sterile medium (Hope Bio-Technology, Qingdao, China). The plants were incubated at 25 °C with a photoperiod of 14 h of light and 10 h of darkness, and the plants could be used for infection when they bore 4–6 true leaves.

### 4.2. Treatment of I. laevigata under Salt Stress and Drought Stress

To further verify whether *IlWRKY70* is involved in the responses of *I. laevigata* to drought and salt stresses, one-year-old *I. laevigata* plants with consistent growth were selected for water culture for seven days. They were treated with NaCl at a concentration of 150 mM and PEG-6000 at a concentration of 20%. The real-time expression level of *IlWRKY70* in the leaves was detected after 0, 1, 3, 6, 12, and 24 h.

### 4.3. IlWRKY70 Gene Cloning and Phylogenetic Tree Analysis

The total RNA was extracted from the roots of *I. laevigata* using the Plant RNA Kit (OMEGA). The integrity of the total RNA was assessed through agarose gel electrophoresis, and high-quality RNA was subsequently reverse-transcribed into complementary DNA (cDNA) using the Primer Script TM RT Reagent Kit (TaKaRa, Beijing, China). In accordance with the sequence previously annotated as *WRKY70* (designated as *IlWRKY70*) in the root transcriptome of *I. laevigata* in our prior research, cloning primers were designed using Primer Premier 5. The obtained primer sequences were as follows: 5′-ATGGAAGGTACCAAAACTGG-3′ and 5′-ATAGAGAGTTCCTCCTTGATTGTAATC-3′. The sequences were cloned using the KOD-Plus-Neo Kit (ToYoBo, Shanghai, China), with an annealing temperature set at 56 °C. Subsequently, the cloned sequences were inserted into the Cloning vector pEASY^®^-Blunt Zero Cloning Kit (TransGen, Beijing, China) and transformed into *Escherichia coli* DH5α (WEIDI). WRKY70 protein sequences from various species were downloaded from the National Center for Biotechnology Information (NCBI) website (https://www.ncbi.nlm.nih.gov/, accessed on 20 July 2022). Multiple sequence alignment was conducted using DNAMAN (Version 5.2.2), and phylogenetic analysis was carried out using MEGA 5.0 (Version 10.1.8). To analyze the physicochemical properties of *Il*WRKY70, the ExPASy website’s ProtParam tools (https://web.expasy.org/protparam/, accessed on 20 July 2022) and ProtScale tools (https://web.expasy.org/protscale/, accessed on 20 July 2022) were employed. The experimental procedures followed the instructions provided with the respective kits, and the primers were synthesized by RuiBiotech Inc. (China, Harbin, China)

### 4.4. Expression Vector Construction

The pCAMBIA1300:*IlWRKY70*-*GFP* carrier was constructed for heterologous expression by homologous recombination. In the first step, SnapGene (version 4.2.4) was used to design homologous recombination primers based on the target fragment and the plant vector sequence, and the SalI and BamHI restriction sites were inserted. In the second step, the following homologous recombination primers for *IlWRKY70* were used: 5′-TTGATACATATGCCCGTCGACATGGAAGGTACCAAAACTGG-3′ and 5′-CCCTTGCTCACCATGGATCCATAGAGAGTTCCTCCTTGAT-3′, underscores represent homology arms. With positive plasmid cloning as the template, the target fragment with the homology arm was amplified using a KOD-Plus-Neo Kit (ToYoBo, Shanghai, China), and the annealing temperature was 63 °C. Next, SalI and BamHI enzymes (TaKaRa, Beijing, China) were selected for the linearization of the plant vector, and the linearized product was recombined with the homology arm amplification product using ClonExpressII One-Step Cloning Kit (Vazyme, Nanjing, China). The recombinant pCAMBIA1300-*IlWRKY70*-*GFP* was transformed into *E. coli* DH5α (WEIDI, Shanghai, China) for sequencing and preservation. Finally, the positive pCAMBIA1300-*IlWRKY70*-*GFP* plasmid obtained by sequencing was transferred into *A. tumefaciens* GV3101 (WEIDI, Shanghai, China) for subsequent infection experiments. The experimental methods were according to the instructions for the use of each kit. Primer synthesis and sequencing services were provided by RuiBiotech Inc. (Harbin, China)

### 4.5. Subcellular Localization

Using the gene gun (PDS-1000) method [40], the pCAMBIA1300:*IlWRKY70*-*GFP* and empty-vector pCAMBIA1300:GFP plasmids were transiently transformed into onion epidermal cells, and the nuclei were stained with DAPI dye [41]. After 24 h of incubation in MS medium at 25 °C in the dark, the GFP expression in cells was observed using a fluorescence microscope (Leica) excited at 395 nm and DAPI nuclear localization dyestuff excited at 358 nm.

### 4.6. Establishment of Transgenic Lines

Agrobacterium GV3101 strains harboring the pCAMBIA1300:*IlWRKY70-GFP* plasmid and the empty-vector pCAMBIA1300*-GFP* plasmid were activated, and transgenic lines were generated through the leaf-disc method mediated by *A. tumefaciens* [42]. Plant genomic DNA from the transformed plants served as a template and was extracted using the plant genomic DNA extraction kit (TianGen, Beijing, China). PCR-positive plants were identified using *IlWRKY70* homologous-arm recombinant primers, as described in the previous vector construction step. T0 generation plants were selected on an MS medium supplemented with 25 mg/L Hygromycin B and confirmed via PCR. Subsequently, seeds from inbred lines were meticulously chosen in successive generations until the T3 generation. Three transgenic lines (named OE-1, OE-2, and OE-3) and one empty-vector line (EV) were randomly selected for further experiments.

### 4.7. Treatment of Transgenic N. tabacum under Salt Stress and Drought Stress

To investigate the salt and drought tolerances of *IlWRKY70*-overexpressing tobacco lines in comparison with control lines, the seeds and seedlings of the transgenic plants were treated with salt and drought stresses, respectively, and the stress conditions were set according to related studies [19,43]. During the seed-stress treatment, wild tobacco seeds and T3 seeds from transgenic and empty-vector lines were sown on MS media containing varying concentrations of NaCl (0, 100, 150, and 200 mM) and mannitol (0, 100, 200, and 300 mM). These seeds were placed in an environment at a temperature of 26 °C, subjected to 16 h of light and 8 h of dark cycles for a duration of 15 days, and then monitored for any observable phenotypic changes. The samples designated for the total root length measurements were sown in square Petri dishes under identical stress conditions and were oriented vertically to ensure vertical root growth, facilitating the convenient measurement of the total root length [42]. In the seedling-stress treatment, the wild-type, transgenic, and empty-vector positive plants were first grown for 30 days at 28 °C and a relative humidity of 75% under 16 h of light and 8 h of darkness, and the cultivation medium comprised peat soil, perlite, and vermiculite mixed in a 3:1:1 ratio. Subsequently, plants in the same growth stage were selected for either salt-stress or drought-stress treatments. For the salt-stress treatment, a 100 mL solution of NaCl at a concentration of 300 mM was applied to the cultivation substrate every three days. As for the drought treatment, it involved in subjecting the plants to natural drought conditions for a span of 14 days. Phenotypic observations and physiological data measurements were conducted on days 0, 7, and 14.

### 4.8. Phenotypic Observation and Physiological Data Measurement of Transgenic Plants under Salt and Drought Stresses

To ensure the accuracy of the data, three plants were selected from each line of each treatment gradient for measurements which were repeated three times. After 15 days of treatment, the germination rate, total root length, and fresh weight of the seeds under the stress treatments were observed and recorded. Total root length data were obtained using a root scanner (Epson Expression 11000XL, Epson, Beijing, China). For the seedlings under the stress treatments, data were measured at 0, 7, and 14 days of their stress cycle. The net photosynthetic rate, stomatal conductance, intercellular CO_2_ concentration, and transpiration rate of the plants were measured using a portable photosynthesis meter (LI-6400, ecotek, Beijing, China). The total chlorophyll content was measured using acetone extraction [44]. The second fully expanded leaf of the upper part of the plant was selected, and the leaves were cut off after being treated with tin foil for 30 min in the dark. The chlorophyll fluorescence data were measured using a portable modulated chlorophyll fluorometer (PAM-2500, WALZ, Effeltrich, Germany) [42]. The content of malondialdehyde (MDA), the activity of superoxide dismutase (SOD), and the activity of peroxidase (POD) were determined according to the research method of Wang [43]. The catalase (CAT) activity was measured using the catalase assay kit G0105F (Grace Biotechnology, Shanghai, China), and the hydrogen peroxide content was measured using the hydrogen peroxide assay kit G0112F (Grace Biotechnology, Shanghai, China). The superoxide anion content was measured with the use of the superoxide anion kit G0116F (Grace Biotechnology, Shanghai, China) according to the method provided in the instructions of the kit. The stressed tobacco leaves were cut into leaf discs with a diameter of 1.5 cm, referring to the method of Wang [43], and p-nitro-blue tetrazolium chloride (NBT) and 3,3′-diaminobenzidine tetrahydrochloride (DAB) stainings were used to detect the contents of superoxide anions and hydrogen peroxide in the leaves, respectively.

### 4.9. Reverse Transcription PCR (RT-PCR) and Real-Time Quantitative PCR (RT-qPCR)

The expression patterns of *IlWRKY70* in different parts of *I. laevigata* were measured from roots, tubers, and leaves of normal growth, and the responses of *IlWRKY70* in *I. laevigata* were measured at 0, 1, 3, 6, 12, and 24 h after salt and drought stresses. Tobacco leaves with a positive overexpression of *IlWRKY70* at 30 days of growth were used to measure the expression of *IlWRKY70*. The expression levels of stress-response genes (*NtSOD*, *NtCAT*, and *NtPOD*) were measured in WT, EV, and three overexpressed *IlWRKY70* lines of tobacco leaves at 0, 7, and 14 days of NaCl and drought stresses. The method of the total RNA extraction and reverse transcription to cDNA is the same as the method of the total RNA extraction and reverse transcription above. The *IlPP2A* and *NtTUBA* genes are the internal reference genes of *I. laevigata* and tobacco, respectively. Real-time quantitative PCR was performed using the SYBR Green I method. The specific experimental procedures were followed according to the instructions of the UltraSYBR Mixture (CWBIO, Beijing, China). The results were calculated as 2^−ΔΔCT^. The quantitative primers were as follows: *IlPP2A*, 5′-TCGCATCAAGACAGGAGAAG-3′ and 5′-GGGAATGAGAAGGGAAGAAT-3′; *NtTUBA*, 5′-CTCCTATGCTCCTGTCATTTC-3′ and 5′-GGCGAGGATCACACTTAAC-3′; *IlWRKY70*, 5′-CGCAACTCGGGGCAATATTG-3′ and 5′-CACTGCTGACTACGTCCTCG-3′; *NtSOD*, 5′-CTCCTACCGTCGCCAAAT-3′ and 5′-GCCCAACCAAGAGAACCC-3′; *NtPOD*, 5′-CCTCAGCTTCAAGCATTATGTCCA-3′ and 5′-ACCTTTGTAGAAGCATCGGTCCAC-3′; *NtCAT*, 5′-AGGTACCGCTCATTCACACC-3′ and 5′-AAGCAAGCTTTTGACCCAGA-3′.

### 4.10. Statistical Analyses

Excel (Version 2021) was used for data recording and statistic analysis. The least significant difference method was used to test the data in the confidence interval (*p* < 0.05), data analysis was performed using SPSS (Version 26), and Origin (Version 2021) was used for drawing graphs.

## 5. Conclusions

In summary, *IlWRKY70* plays a significant role in the responses of *I. laevigata* to salt and drought stresses. The overexpressionof *IlWRKY70* can strengthen the abilities of tobacco seeds and seedlings to resist salt stress and drought stress; enhance the seed germination rate, total root length, and fresh weight; relatively raise the level of photosynthesis in seedlings under salt stress and drought stress; reduce the plant’s stomatal conductance to preserve the plant’s moisture contents; and enhance the antioxidant enzyme system, which makes tobacco more resistant to salt and drought stresses. This study provides insight into important functional genes for salt- and drought-tolerance breeding in iris plants, laying the foundation for revealing the salt- and drought-tolerance mechanisms of *I. laevigata*.

## Data Availability

Data are contained within the article.
